# Noninferiority Study Comparing the Efficacy and Safety of a New Hyaluronic Acid (HA) Filler Containing Lidocaine With an Existing HA Filler for the Treatment of Nasolabial Fold Wrinkles: A Randomized, Double‐Blind, Split‐Face Trial

**DOI:** 10.1111/jocd.70309

**Published:** 2025-07-07

**Authors:** Ji Young Cho, Seung Hyeon Kim, Hyesoo Cho, Yoonjae Park, Hee Joo Yang, Jee Soo An, Chong Hyun Won, Jong Hun Lee

**Affiliations:** ^1^ Department of Dermatology, Asan Medical Center University of Ulsan College of Medicine Seoul Korea; ^2^ Department of Plastic and Reconstructive Surgery, Nowon Eulji Medical Center, School of Medicine Eulji University Seoul Korea; ^3^ Department of Plastic and Reconstructive Surgery, Sanggye Paik Hospital, School of Medicine Inje University Seoul Korea

**Keywords:** dermal fillers, hyaluronic acid, nasolabial fold

## Abstract

**Background:**

The aesthetic use of hyaluronic acid (HA) has led to the development of various commercial HA fillers. Crosslinked HA dermal fillers are commonly used to treat facial wrinkles. This study demonstrated the non‐inferiority of the newly developed hyaluronic acid filler by comparing its efficacy and safety to that of the existing hyaluronic acid filler.

**Methods:**

This 48‐week study was designed as a randomized, double‐blind, split‐face trial. Subjects who met the inclusion and exclusion criteria had a total of four follow‐up visits after receiving a dermal filler in the nasolabial folds. Efficacy was assessed using the Wrinkle Severity Rating Score (WSRS) and the Global Aesthetic Improvement Scale (GAIS). Safety was determined through the evaluation of laboratory tests, monitoring adverse events, and verifying vital signs.

**Results:**

WSRS measured at week 24 of the follow‐up visit showed no statistically significant difference, with scores of 2.02 ± 0.71 in the new filler group and 2.27 ± 0.68 in the existing filler group. Even at the 48‐week follow‐up visit, the improvement rate in the Wrinkle Severity Rating Scale (WSRS) of more than 1 point did not show a statistically significant difference between the new filler group and the existing filler group. The GAIS scores at each follow‐up visit showed no statistically significant differences between the two groups. The most frequently reported symptoms related to the test device during the 48‐week trial were pain and swelling. However, these symptoms were not statistically significant when compared to the control group from a treatment perspective.

**Conclusions:**

In a 48‐week clinical trial aimed at treating nasolabial fold (NLF) wrinkles, the new hyaluronic acid (HA) filler demonstrated efficacy and safety comparable to that of existing control filler. As a result, the new HA filler is both an effective and safe option for improving NLF wrinkles.

**Trial Registration:**

This study was registered with ClinicalTrials.gov under the registration number NCT06310863

## 1 Introduction

One of the things you can't avoid is wrinkles with age. Although wrinkles can be said to be a mark and a decoration of our lives, sometimes wrinkles are also a nuisance that makes us look presbyopic. In particular, the nasolabial folds (NLFs), which are located in the center of the face, are considered to be one of the big factors that influence the impression, and many people suffer from NLFs [1, 2].

Methods for treating nasolabial fold (NLF) wrinkles include thread lifts, Botox, and dermal filler treatments. Appropriate treatment methods depend on the cause of NLF wrinkles. The NLF wrinkles occur when the epidermis thins, the subcutaneous fat layer decreases, and there is a loss of elastic fibers and collagen, along with a weakening of the basal muscles. Recently, the method that has been attracting attention is filler injection. Filler injection is a procedure in which fillers similar to the body's components, such as hyaluronic acid, are injected into areas that are deeply dug or lack volume. This study is conducted using fillers to improve NLF wrinkles. In the case of NLF fillers, harmless ingredients are injected into the wrinkled areas to improve wrinkles or fill in the areas lacking volume. There are various types of fillers, including collagen, hyaluronic acid (HA), poly‐L‐lactic acid (PLLA), calcium hydroxylapatite, and silicone, each of which has distinct ingredients, functions, effects, and retention periods depending on the injection site and the specific filler used [3].

Among these fillers, hyaluronic acid fillers are linear polysaccharides found in all animals. They are essential components of extracellular matrices and can bind to water up to 1000 times their own weight, making them the most commonly used dermal filler for wrinkle correction [4]. However, a disadvantage of natural hyaluronic acid is its rapid decomposition, with a half‐life of approximately 24 h. To overcome this limitation, a viscoelastic polymer was developed that utilizes a crosslinking agent, such as BDDE, to create chemical crosslinking. Cross‐linked hyaluronic acid exhibits improved resistance to enzymatic degradation and has a longer duration of effect compared to natural hyaluronic acid [5, 6, 7].

Restylane Lyft, which is chemically crosslinked HA and contains lidocaine, is a representative filler that reduces pain during filler injection and is widely used as a control test device for many newly produced dermal fillers. This study aimed to confirm that the newly developed LASBEAU Strong (ExocoBio Inc., Seoul, Korea) is not inferior in efficacy and safety compared to Restylane Lyft, which is chemically cross‐linked and contains lidocaine for pain relief.

## 2 Materials and Methods

### 2.1 Subjects

This study was conducted at two centers in South Korea: Asan Medical Center and Nowon Eulji Medical Center. From November 2019 to June 2020, we enrolled 72 healthy subjects over the age of 19 with a WSRS score of 3 or 4 and seeking temporary correction in NLFs.

This study excluded individuals with hemorrhagic disorders or those who had received calcium hydroxyapatite (CaHA) or poly‐L‐lactide (PLA) filler therapy within the past year. Additionally, participants who had applied topical formulations to their faces (excluding drug‐grade steroids, retinoids) within 4 weeks prior to the screening date or during the clinical trial period were also excluded. Other exclusions included individuals who had undergone anti‐wrinkle therapy, acne scar treatment, or plastic surgery (including botulinum toxin injections) within the last 24 weeks, subjects who had received permanent skin extension implants such as soft foam or silicone, those with skin diseases, individuals with a history of facial wound infections, keloids, or thick scars, as well as pregnant individuals or those likely to become pregnant.

### 2.2 Materials

LASBEAU Strong (ExocoBio Inc., Seoul, Korea) as a test filler is a transparent colorless liquid filler consisting of 24 mg/mL cross‐linked hyaluronic acid that was used as a test filler. LASBEAU Strong was administered via sterile, 1.0‐mL, prefilled syringes with 26gauge needles containing 1,4‐butanediol diglycidyl ether (BDDE) and lidocaine HCl.

Restylane Lyft (Galderma Korea Inc., Seoul, Korea) is a sterile gel filler made from hyaluronic acid derived from Streptococcus bacteria, crosslinked with BDDE and stabilized at 20 mg/mL with lidocaine. It is injected using 1.0‐mL prefilled syringes with 29‐gauge needles.

The rheological properties (specifically G′, G″, complex modulus, tan delta, viscosity, concentration) of the two fillers are presented in Table 1.

**TABLE 1:** Rheological properties at 0.1 Hz (*T* = 34°C).

	G′ (Pa)	G″ (Pa)	Complex modulus	tan delta (G″/G′)	Viscosity (cP)	HA conc. (mg/mL)
Test filler	230.5	39.7	235.3	0.172	2 098 000	24
Conventional filler (Restylane Lyft)	605.6	141.5	622.2	0.234	1 230 000	20

In terms of viscosity among the rheological properties, the test filler exhibited higher values than the conventional filler. Consequently, a 26‐gauge needle provides similar injection pressures and ensures the smooth delivery of filler material in both groups. This choice helps standardize our trial methods regardless of each filler's viscosity.

### 2.3 Treatment

All subjects' faces were digitally photographed at each visit. The subjects were randomly assigned using a random‐number table to determine which NLF on the right or left would receive any filler.

Subjects and researchers are double‐blind, with all identifying information removed from the photograph.

The treatments were performed by skilled physicians(investigators). Each physician followed the standardized protocol to ensure uniformity in the injection technique and was instructed to apply the same injection technique for both products. The injection technique used retrograde linear threads, where the needle is inserted and retracted while applying consistent pressure to create a linear filler deposit. This method is effective for filling folds and fine lines, and all subjects received injections without anesthesia. At the initial treatment, a maximum volume of 1.0 mL was administered for each NLF.

### 2.4 Efficacy Evaluation

The mean value in the WSRS scores evaluated by three independent investigators from baseline to 24 weeks was utilized as the primary efficacy measure (Table 2). WSRS scores were assessed by three independent investigators and were approved only if all scores were consistent. In cases where the WSRS scores differed among the investigators, the independent investigators conducted a reevaluation and agreed upon a unified score.

**TABLE 2:** WSRS (Wrinkle Severity Rating Scale).

Grade	Notes
1	No flattening of the upper lip
2	Mild flattening of the upper lip
3	Moderate flattening of the upper lip, mild wrinkle mainly due to volume loss
4	Moderate wrinkling, moderate lengthening of the distance between nose and lip border due to volume loss, some yellowing and sun damage
5	Severe wrinkling and wizened appearance, marked lengthening of the distance between nose and lip border due to volume loss

WSRS and GAIS scores were evaluated at each follow‐up visit and were used as secondary efficacy assessments. Additionally, an independent investigator assessed the proportion of subjects with one or more improved WSRS scores at weeks 24 and 48 (Table 3). The subject's photograph taken before the filler injection at baseline served as a reference image to evaluate the improvement of nasolabial folds (NLFs) after the injection. During the telephone visit at week 36, participants' adverse events (AEs) and concomitant medications were reviewed. Furthermore, during the follow‐up visit at week 48, the filler injection site was photographed, and WSRS or GAIS evaluations were conducted.

**TABLE 3:** GAIS (Global Aesthetic Improvement Scale).

Grade	Notes
3	Very much improved
2	Much improved
1	Improved
0	No change
−1	Worse

### 2.5 Safety Evaluation

AEs and serious adverse events (SAEs) were presented as numbers of subjects, percentages, and incidence.

### 2.6 Sample Size

According to the FDA's clinical review of Restylane [8], the mean ± standard deviation of WSRS evaluated by the investigators at week 24 was 2.36 ± 0.78 in Restylane and 2.94 ± 0.76 in the past control group, and the difference between the application groups (Restylane‐past control group) was 0.58. Based on the results of this study, the non‐inferiority limit was set at 0.29, which is 50% of 0.58. The standard deviation of WSRS was assumed to be 0.78, the largest standard deviation, and the difference between the test group and the control group was assumed to be zero.

- Non‐inferiority clinical trial
- Hypothesis: 𝐻0∶ 𝜇𝐷 ≥ 𝛿 𝑣𝑠. 𝐻1∶ 𝜇𝐷 < 𝛿
- $n=\frac{{\left(\;\mathrm{Ζ\alpha}+\mathrm{Z\beta}\right)}^{2}\times \mathrm{\sigma D}2^{2}}{{\left(\mathrm{\mu D}-\delta \right)}^{2}}=\frac{{\left(1.96+0.842\right)}^{2}\times 0.78^{2}}{0.29^{2}}=56.80≅57$
- One‐sided α level: *α* = 0.025
- Power of the test: 1‐*β* = 0.8
- The mean difference of WSRS: 𝜇𝐷 = 0
- The largest standard deviation of WSRS: 𝜎𝐷 = 0.78
- The non‐inferiority limit: *𝛿* = 0.29

Based on the above calculation evidence, the minimum number of subjects required for a one‐sided significance level of 2.5% and a power of 80% is 57. Considering the dropout rate of 20, a total of 72 subjects were recruited.

### 2.7 Statistical Analysis

The efficacy evaluation analysis utilized the two‐sample test, Wilcoxon rank‐sum test, chi‐square test, and Fisher's exact test. The comparisons were analyzed using two‐tailed tests with a significance level of 5%. We applied the paired *t*‐test, Wilcoxon signed‐rank test, and McNemar's test to evaluate the laboratory test results, vital signs, and physical examinations.

## 3 Results

### 3.1 Subjects and Efficacy Outcomes

**FIGURE 1:**
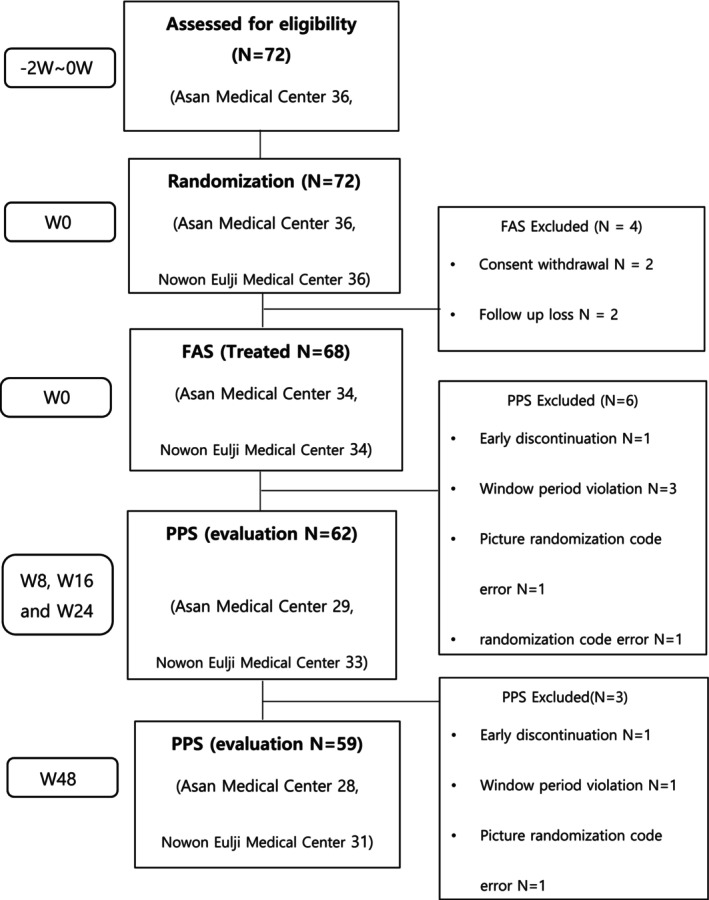
Flowchart of subjects included in the trial.

A total of 72 subjects agreed to participate in this trial and all were randomized to receive at least one HA filler injection. Four subjects were excluded from the safety set: two participants withdrew consent and two failed follow‐up. An analysis was performed on 62 subjects in the PPS, as there was one drop‐out, three violations of the window period, one random assignment code error, and one photo randomization code error. In the long‐term safety evaluation, 59 subjects were further analyzed, excluding three subjects by long‐term safety evaluation in the PPS: one drop‐out, one window period violation, and one error in photo randomization code. A representative image of the subjects is shown in Figure 1.

#### 3.1.1 Primary Efficacy Endpoint

**FIGURE 2:**
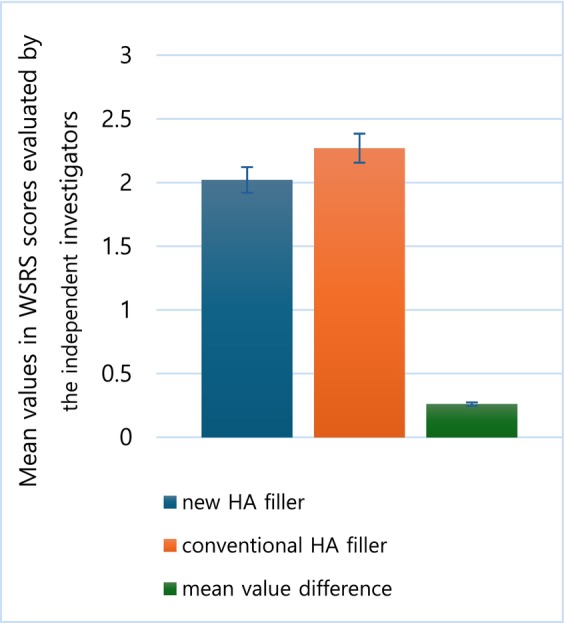
The mean value of WSRS score evaluated by independent investigators at week 24.

**FIGURE 3:**
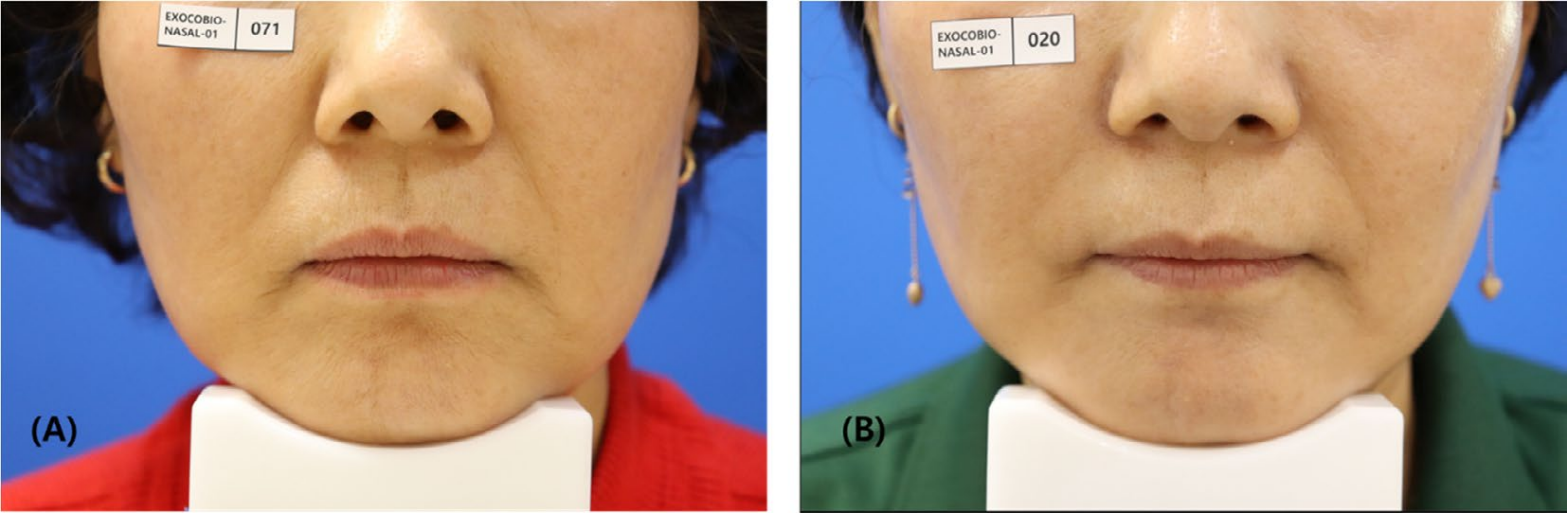
Aesthetic outcomes in a 45‐year‐old female subject. (A) At baseline, (B) at week 24 following the HA filler injections. The subject's left mid‐face was injected with the new HA filler containing lidocaine and right mid‐face with the conventional HA filler.

The mean value of WSRS scores for both groups, assessed by independent investigators at week 24 following filler injection, served as the primary measure of efficacy. The mean value of WSRS score in the new filler group was 2.02 ± 0.71, whereas the mean value of WSRS score in the existing filler group was 2.27 ± 0.68. The mean value difference between the filler injection groups at week 24 was −0.26 ± 0.69 (97.5% one‐sided confidence interval by *t*‐distribution). The non‐inferiority margin established in the study was 0.29; however, the new filler group did not demonstrate inferiority compared to the existing filler group. This conclusion is supported by the upper limit of the one‐sided 97.5% confidence interval, which was −0.01 (Figure 2). The corresponding results, along with clinical photos, are presented in Figure 3.

#### 3.1.2 Secondary Efficacy Endpoint

**FIGURE 4:**
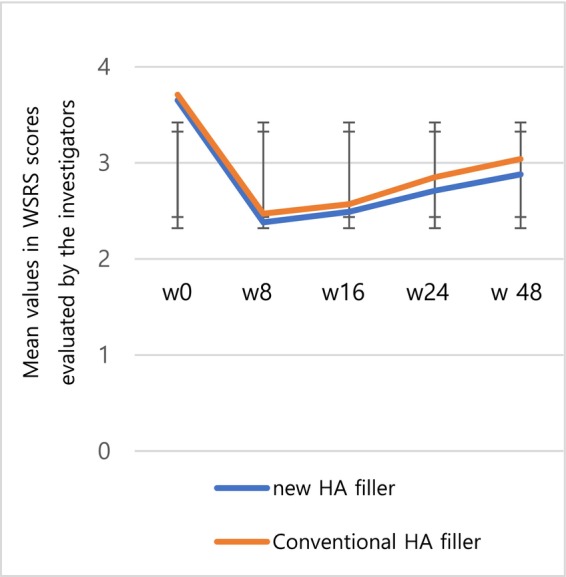
The mean value of WSRS scores in the test group and the control group.

The secondary efficacy endpoints were assessed using the mean values of the WSRS and GAIS taken at each follow‐up visit. The mean values of the WSRS were as follows: 2.38 ± 0.75 at week 8, 2.49 ± 0.86 at week 16, 2.71 ± 0.90 at week 24, and 2.88 ± 0.94 at week 48 for the test group, and 2.47 ± 0.68 at week 8, 2.57 ± 0.78 at week 16, 2.85 ± 0.83 at week 24, and 3.04 ± 0.87 at week 48 for the control group. No visits demonstrated a statistically significant difference (Figure 4).

**FIGURE 5:**
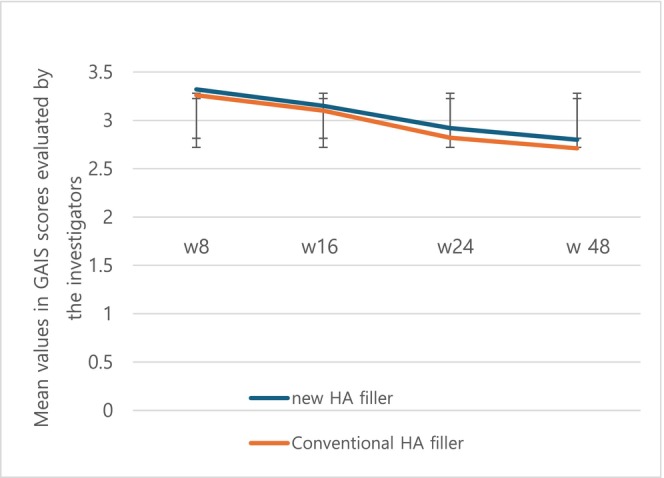
The mean value of GAIS scores in the test group and the control group.

Similarly, the mean values of the GAIS were as follows: 3.32 ± 0.67 at week 8, 3.15 ± 0.87 at week 16, 2.92 ± 0.82 at week 24, and 2.80 ± 0.83 for the test group. In comparison, the control group exhibited mean values of 3.26 ± 0.57 at week 8, 3.10 ± 0.80 at week 16, 2.82 ± 0.76 at week 24, and 2.71 ± 0.79 at week 48. No visits demonstrated a statistically significant difference (Figure 5).

**FIGURE 6:**
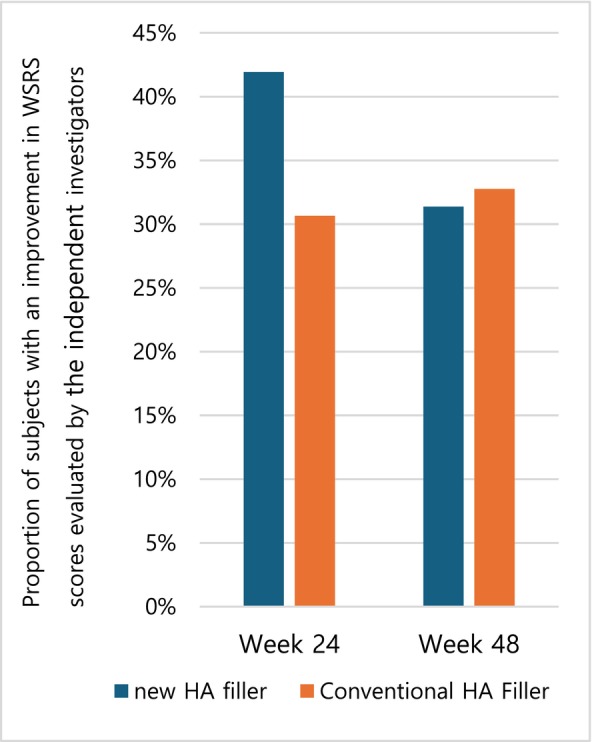
The proportion of subjects with improved WSRS scores at weeks 24 and 48.

A secondary efficacy endpoint was the proportion of subjects with independently evaluated WSRS scores improving by at least one point at weeks 24 and 48. In the new filler group, 41.94% (26 subjects) improved, while 30.65% (19 subjects) improved in the existing filler group. At week 24 and at week 48, the proportions were 31.37% (16 subjects) in the test group and 32.76% (19 subjects) in the control group. There was no statistically significant difference between the two groups (Figure 6).

#### 3.1.3 Safety Outcomes

There were no systemic AEs related to the investigational test device reported at weeks 24, 36, and 48. Systemic SAEs reported during the trial were limb injury and neurological injury, which were not related to the investigational devices, and all patients recovered. Overall, the most reported symptoms in the two groups were pain (41.67%) and swelling (40.28%) (Table 4) [9]. Despite this, pain and swelling related to the intervention were neither dominant nor statistically significant (*p* = 0.1614 and *p* = 0.1168 respectively).

**TABLE 4:** Local adverse events in the two groups.

AE	Test group		Control group		*p*
Pruritus	13	[18.06]	10	[13.89]	0.4950*
Bruise	17	[23.61]	17	[23.61]	1.0000*
Swelling	29	[40.28]	21	[29.17]	0.1614*
Tenderness	12	[16.67]	11	[15.28]	0.8221*
Pain	30	[41.67]	21	[29.17]	0.1168*
Erythema	18	[25.00]	14	[19.44]	0.4227*
Other	7	[9.72]	6	[8.33]	0.7712*

*Note:* Values are presented as number [%].

## 4 Discussion

As public interest in improving the volumetric appearance of facial wrinkles grows, numerous treatment methods are being developed to increase the range of available options, including fillers and autologous fat transplants. The presence of pronounced nasolabial wrinkles is a readily perceptible indicator of facial aging, prompting individuals to seek more proactive treatment options.

Consequently, The non‐surgical procedure under consideration is filler injection. We aim to develop a minimally invasive, painless filler that effectively maintains volume. Each filler has unique properties in terms of viscosity, durability, efficacy, and safety, and HA among fillers is a hygroscopic molecule that can bind to water at 1000 times its volume [10, 11]. Furthermore, HA is a highly suitable material for use as a skin filler, as the risk of triggering immune response and implant rejection may be negligible due to interspecies similar ubiquitous chemical structures [12]. Other features that may contribute to the popularity of HA filler treatment include ease of administration, minimum recovery time, and immediate outcome [10].

Filler injection techniques range from simple linear threading and small aliquot deposition to complex methods like serial linear threading and radial fanning. We used retrograde linear threading to correct each nasolabial fold (NLF), which is effective for subcutaneous and dermal filling of folds and fine lines.

This study investigated the effects of the new HA filler which is a colorless, transparent liquid containing 24 mg/mL of cross‐linked HA, on the improvement of nasolabial folds. The investigation employed a split‐face, double‐blind methodology to compare the outcomes with those achieved using an existing HA filler. Our findings demonstrate that the new hyaluronic acid (HA) filler is not inferior to the existing HA filler in treating nasolabial folds. This conclusion is supported by comparable scores on the Wrinkle Severity Rating Scale (WSRS), the Global Aesthetic Improvement Scale (GAIS), and the proportion of subjects showing improvement in WSRS scores by week 48. Consequently, we conclude that, at least up to week 48, the novel HA filler demonstrates efficacy comparable to that of the conventional HA filler.

Regarding safety, the most common AEs in both the test and control groups were pain and swelling [9]. Limb injuries and nervous system injuries were reported as SAEs but were not related to either filler, and all participants recovered without complications. No clinically significant differences in laboratory test results or vital signs were observed between the test and control groups. The new hyaluronic acid (HA) filler was well tolerated, with no significant side effects noted up to 48 weeks. This study investigated the efficacy and safety of hyaluronic acid fillers over an extended period of 48 weeks, in contrast to the recent 24‐week studies [13, 14, 15, 16, 17]. The results demonstrated that the new hyaluronic acid (HA) fillers are not inferior to conventional HA fillers in providing temporary improvement of nasolabial folds and in terms of long‐term safety.

However, delayed‐onset AEs, such as the formation of foreign body granulomas or inflammation, have been documented in the literature and may potentially occur [18]. Therefore, additional studies involving a larger number of subjects and an extended follow‐up period are necessary to confirm the long‐term safety of the novel hyaluronic acid (HA) filler.

In conclusion, both types of fillers can be considered stable procedures. Therefore, new HA fillers may represent a suitable option for ensuring safety and efficacy in the non‐surgical treatment of nasolabial folds.

## Author Contributions

J.Y.C., S.H.K., Y.P., C.H.W., and J.H.L. performed the research. C.H.W. and J.H.L. designed the research study. H.C., H.J.Y., and J.S.A. contributed essential reagents or tools. H.C. and H.J.Y. analyzed the data. J.Y.C., S.H.K., and Y.P. wrote the paper. All authors have read and approved the final manuscript.

## Ethics Statement

The study was approved by independent ethics committees, conformed to the Declaration of Helsinki, and was conducted in compliance with Good Clinical Practice. Subjects provided signed informed consent for participation.

## Conflicts of Interest

The authors declare no conflicts of interest.

## Data Availability

The data that support the findings of this study are available from the corresponding author upon reasonable request.
